# Residual Stress in Friction Stir Welding of Dissimilar Aluminum Alloys: A Parametric Study

**DOI:** 10.3390/ma18020316

**Published:** 2025-01-12

**Authors:** Zulqarnain Sarfaraz, Yasser Riaz Awan, Hasan Aftab Saeed, Rehan Khan, Michał Wieczorowski, Naveed Akmal Din

**Affiliations:** 1 Department of Mechanical Engineering (CEME), National University of Sciences and Technology (NUST), Sector H-12, Islamabad 46000, Pakistan; zsarfaraz.me19ceme@student.nust.edu.pk (Z.S.); yasser@ceme.nust.edu.pk (Y.R.A.);; 2 Mechanical Engineering Department, University of Engineering and Technology, Taxila 47050, Pakistan; 3 Faculty of Mechanical Engineering, Institute of Applied Mechanics, Poznan University of Technology, 3 Piotrowo St., 60-965 Poznan, Poland; michal.wieczorowski@put.poznan.pl

**Keywords:** friction stir welding, thermomechanical model, dissimilar joints, aluminum alloys, residual stress

## Abstract

Welding-induced residual stress has the capacity to significantly compromise the integrity of mechanical components. Its minimization therefore plays a critical role in the selection of process parameters during the welding process. Friction stir welding is a useful joining technique to weld many materials that are not amenable to the traditional welding techniques. Using a sequentially coupled thermomechanical three-dimensional finite element simulation, this work aimed to quantitatively evaluate the influence of the tool rotational and traverse speeds on the generation of residual stress in the friction stir welding of dissimilar aluminum alloys AA2024−T3 and AA5086−O. The model was validated using established experimental and numerical results. The procedure entailed an initial thermal analysis, the results of which were superposed on a mechanical model to determine the distribution of the residual stress across the welded alloy. The results showed that longitudinal residual stress was dominant as compared to lateral stress. It was also demonstrated that, although the tool rotational speed and the tool traverse speed both affected the post-weld temperature distribution and consequently the longitudinal residual stress, the influence of the former was more substantial. Furthermore, the peak values of the residual stress were found on the retreating side (AA5086−O), making it more critical for the selection of welding process parameters.

## 1. Introduction

The need to join dissimilar materials has been prompted by the perpetual demand for increased efficiency and decreased weight in various industrial sectors. This has resulted in creative solutions that blend the distinctive qualities of several materials to maximize the performance while reducing the overall weight [1]. By combining different materials, industries like the automotive, marine, and aerospace fields create hybrid components that have improved structural performance and a lightweight design [2]. Due to their light weight, aluminum alloys are often utilized as structural elements in these sectors [3,4]. Although traditional techniques like mechanical fastening and adhesive bonding are frequently employed, they have several drawbacks, including increased weight, difficulties in automation, and corrosion-related issues [5,6]. On the other hand, conventional fusion welding is not commonly used to combine incompatible or dissimilar aluminum alloys because their melting points differ, resulting in uneven heating and cooling throughout the welding process. This can cause cracks and faults in the welded junction, compromising its strength and integrity. Other factors that present a significant challenge in fusion welding are the high thermal expansion factor, solidification distortion, and large differences in the solidification temperatures. These factors render dissimilar aluminum alloys highly vulnerable to hot cracking during the fusion welding process [7].

To circumvent these problems, new joining techniques have been developed. One of these is friction stir welding (FSW), which was pioneered by TWI in 1991 [8]. Unlike the traditional welding techniques, FSW is categorized as a solid-state joining method since the material being welded is not melted throughout the process [9]. The goal of FSW is to eliminate the problems associated with fusion welding, brazing, and soldering, making it a popular technique for the combination of many incompatible materials [10]. However, FSW is also not without its disadvantages, such as high pressing force requirements, weld gaps, higher costs, and sensitivity to the tool shape and process parameters. This also provides great scope for improvement in the welding capabilities and process [11]. Recent research has taken analytical, experimental, and numerical routes in investigating various strategies that can lead to the optimization of the tool design and process parameters [12].

As an immediate outcome of the uneven heat distribution and cooling during the FSW process, welded materials generate complicated residual stresses [13]. The ability of structures to support loads is reduced by these stresses. The high stresses in the area around the weld joint may also make brittle fractures and fatigue failures more likely [14]. Experimental evaluations of residual stress in conjunction with weld optimization testing have been employed as a solution to this issue in the past [15]. Common methods for the experimental determination of residual stresses are X-ray diffraction [16], the hole-drilling strain gage method [17], and ultrasonic testing [18], each with its merits and demerits. The ultrasonic method is non-destructive and has high precision but is costly. On the other hand, the mechanical method, while comparatively economical, damages the material and is time-consuming. The X-ray method, another non-destructive option, has implications for health [19].

The experimental examination of all pertinent factors, however, is a time-consuming and costly operation. Complementing this approach, numerical simulation utilizing the finite element method (FEM) is also a viable alternative given the current digital computer capabilities [20]. Improvements in computer technology and the capability of numerical approaches like FEM have improved the accuracy of residual stress examination in welded materials [21]. A validated model has the capacity to generate trustworthy information about FSW processes and tools, which should therefore be able to reduce the flaws and holes generated during welding [22].

Weld zone residual stress has been noted to exist in both the longitudinal and transverse directions. According to experimental findings, longitudinal residual stress is proportional to the traverse speed. This is most likely due to the greater heat gradients and less time available for stress release [23]. Using X-ray diffraction, an investigation of the impact of the rotational and traverse speeds on the residual stress in AA2024−T3 and AA6061−T6 showed that a change in the rotational speed was associated with the largest differences in the magnitude of the residual stress. The intensity of the longitudinal residual stress was reduced when the rotational speed was increased. This was strongly related to the rise in heat input and the fall in thermal misfit between the various weld zones [16]. Additionally, researchers have also included the tilting of the tool spindle as one of the parameters that affects the generation of residual stresses [24].

Thermomechanical modeling to quantify the residual stress in the FSW of dissimilar aluminum grades has also been carried out. The stress values in both AA5086−O and AA6061−T6 indicated that the greatest longitudinal stress was higher than the maximum transverse stress. Moreover, the maximum longitudinal compressive stress spread around the rotating pin, whereas the maximum longitudinal tensile stress was generated near the specimen’s edge [25]. Experimental and theoretical investigations into the thermal profiles and residual stresses in the dissimilar FSW of the same alloys revealed that the tool traverse rate primarily affected the distribution of the transverse residual stress, while the tool rotational speed influenced the magnitude of the maximum longitudinal residual stress. An increase in heat input per unit length of the welded joint led to higher residual tensile stress and a larger affected area. Additionally, it was observed that the mechanical constraints imposed by the welding clamps notably affected the residual stress pattern, with the highest residual stress decreasing when the clamps were promptly released [26].

Finite element analysis has also been employed to investigate the thermal and residual stress conditions in the dissimilar FSW of aluminum alloys AA6061−T6 and AA2024−T3. The longitudinal residual stress in the AA6061 part was found to be higher as compared to the AA2024 part. Moreover, the residual stress was reduced with a rise in the tool rotational speed but increased with an increase in the tool traverse speed. It was concluded that the rotational speed of the tool has a greater impact on the residual stress than its traverse speed [27].

The joining of AA2024−T3 and AA5086−O is required in the shipbuilding industry. In an experimental investigation of the FSW of these dissimilar alloys, four combinations of rotational and traverse speeds were tested for strength characterization, followed by the fractography of the specimens [28]. To date, the residual stress generated in the FSW of these alloys has not been investigated, either experimentally or numerically. There is therefore a need to evaluate this stress, as well as the influence of the relevant process parameters. The present study seeks to address this knowledge gap. In summary, a validated finite element model will be used to investigate the influence of the rotational and traverse speeds on the generation of longitudinal residual stresses during the FSW of these alloys, with AA2024−T3 on the advancing and AA5086−O on the retreating side. This information could lead to the selection of appropriate FSW process parameters for these joints.

## 2. Problem Definition and Methodology

In this work, a sequentially coupled thermomechanical model for the FSW of dissimilar aluminum alloys AA2024−T3 and AA5086−O is created for investigation. A heat transfer model is first developed to calculate the temperature fields throughout the specimen. Thereafter, the temperature fields obtained in the first step are used as inputs into a three-dimensional structural model that incorporates material nonlinearity and rate dependence in order to ascertain the residual stress. To validate the accuracy of the models, the results are then compared to established numerical and experimental results. Subsequently, the study delves into exploring the influence of the rotational and traverse speeds on the values of the residual stress in AA2024−T3 and AA5086−O.

### 2.1. Geometry

**Figure 1:**
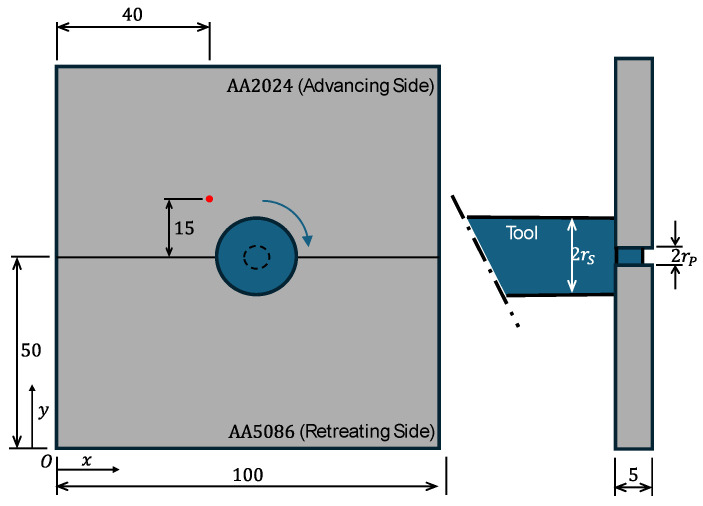
Schematic diagram of plates to be welded (all dimensions are in mm).

Two aluminum plates of AA2024−T3 and AA5086−O are the subjects of this study; they have the following dimensions: 5 mm thickness, 100 mm length, and 50 mm width, as shown in Figure 1. The AA5086−O alloy is selected to be on the retreating side, whereas the AA2024−T3 alloy is on the advancing side. The tool dimensions are ∅20 mm (shoulder), ∅4 mm (threaded pin), and 4.7 mm (height). The downward axial force on the tool is 7 KN.

### 2.2. Thermal Model

During FSW, the transient heat transfer problem necessitates the solution of the heat equation, which can be expressed in the Cartesian coordinate system as [29]

(1) $$k\left(\frac{\partial^{2}T}{\partial x^{2}}+\frac{\partial^{2}T}{\partial y^{2}}+\frac{\partial^{2}T}{\partial z^{2}}\right)+Q_{\mathrm{int}}=c\rho \frac{\partial T}{\partial t}$$

where $Q_{\mathrm{int}}$ is the heat generation due to friction between the tool and workpiece, c is the specific heat capacity, ρ is the material density, k is the thermal conductivity, and T is the absolute temperature. Assuming that the melting point is not reached, it can be expressed as [30]

(2) $$d\dot{q}=\mu p\left(\omega r-U\sin\theta \right)dA$$

where p is the tool local pressure applied to the elemental area dA, and μ is the friction coefficient. The angular velocity ω is 2πN, where N is the number of revolutions per second and r denotes the distance between any point and the tool axis. The tool’s relative speed in relation to the material is represented by the velocity (ωr−Usinθ). The expression demonstrates that the rate of heat production on the advancing and retreating sides differs. If ωr is large enough, then the Usinθ term becomes insignificant in comparison. Thus, to calculate the heat generation at the tool shoulder–workpiece interface, the following formula can be used:

(3) $$Q_{\mathrm{Shoulder}}=\frac{4\pi^{2}}{3}\mu Np\left(r_{S}^{3}-r_{P}^{3}\right)$$

where, $r_{S}$ and $r_{P}$ stand for the shoulder and pin radii, respectively. The heat produced at the contact of the tool–workpiece interface is equivalent to

(4) $$Q_{\mathrm{Pin}}=\frac{4\pi^{2}}{3}\mu Np\left(r_{P}^{3}-3r_{P}^{2}h\right)$$

where h is the pin length. As a result, the total heat produced at the tool–workpiece interface will be equal to

(5) $$Q_{\mathrm{Total}}=\frac{4\pi^{2}}{3}\mu Np\left(r_{S}^{3}-3r_{P}^{2}h\right)$$

These equations assume that the tool pressure p is applied uniformly.

#### Thermal Boundary Conditions

All free surfaces of the workpiece and the tool lose heat to the environment through convection and radiation, while conduction happens through the bottom surface to the supporting plate. The heat loss $q_{h}$ is estimated using Equation (6), taking into account the convection and radiation across each workpiece region apart from the bottom.

(6) $$q_{h}=h_{\mathrm{con}}\left(T-T_{0}\right)+\varepsilon B\left(T^{4}-T_{0}^{4}\right)$$

where T is the workpiece’s absolute temperature, $T_{0}$ is the sink temperature, $h_{con}$ is the convective film coefficient, ε is the emissivity constant, and B is the Stefan–Boltzmann constant. To simplify the analysis, the heat loss from the bottom plate is taken to be

(7) $$q_{b}=h_{b}\left(T-T_{0}\right)$$

where $h_{b}$ represents the convective coefficient of heat loss from the bottom plate. It is assumed that the weld centerline is subject to identical thermal boundary conditions.

### 2.3. Mechanical Model

Given that FSW is a solid-state procedure and does not undergo a phase shift, using the continuum mechanics model, the force equilibrium equation may be written as follows:

(8) $$\int_{s}tds+\int_{V}fdV=0$$

where V stands for the volume and S is the surface enclosing this volume. At a point on S, the Cauchy stress matrix is defined as follows:

(9) t=n·σ

where n is the outward unit normal to S at any point. The differential equation of linear equilibrium is created by applying the Gauss theorem to Equation (8):

(10) $$\left(\frac{\partial }{\partial x}\right)\cdot \sigma +f=0$$

For the purpose of FEM, it is convenient to express the differential equation of equilibrium as an integral equation based on the virtual work principle, which results in

(11) $$\int_{S}t\cdot \delta v\;dS+\int_{V}f\cdot \delta v\;dV=\int_{V}\sigma :\left(\frac{\partial dv}{\partial x}\right)dV$$

where δv is the virtual velocity field.

#### Mechanical Boundary Conditions

The vertical motion of the workpiece is restricted at the bottom surface, which can be expressed as

(12) $${\left(U_{z}\right)}_{z=0}=0$$

The workpiece is also held in position at the ends by clamping. At these clamping points, completely rigid boundary constraints are implemented:

(13) {U}=0

where {U} represents all displacement degrees of freedom. The clamping restrictions are lifted once the weld reaches room temperature. In addition, the displacements along the symmetric surface are taken to be identical.

(14) $${\left[{\left(\left\{U\right\}\right)}_{y=50}\right]}_{5086}={\left[{\left(\left\{U\right\}\right)}_{y=50}\right]}_{2024}$$

## 3. Finite Element Model

A commercial FEA program was used to solve the sequentially coupled thermomechanical model of the FSW process to assess the residual stress. The model was then utilized to evaluate the process parameters for AA2024−T3 and AA5086−O. For this purpose, the full factorial DOE matrix showing the utilized combinations of welding parameters (tool traverse speed and tool rotational speed) is shown in Table 1.

The thermophysical characteristics and temperature-dependent mechanical properties [26,27] that are used in the three-dimensional model are given in Table 2 and Table 3.

The Johnson–Cook plasticity model [31] is employed to take into account the plastic behavior of both materials. This model relates the stress, strain, strain rate, and temperatures using the following relation (with reference strain rate $1.0\;s^{-1}$):

(15) $$\sigma =\left(A+B\varepsilon^{n}\right)\left[1+\mathrm{Cln}\left(1+\frac{\dot{\varepsilon }}{{\dot{\varepsilon }}_{0}}\right)\right]\left[1-{\left(\frac{T-T_{\mathrm{room}}}{T_{\mathrm{melt}}-T_{\mathrm{room}}}\right)}^{m}\right]$$

The Johnson–Cook model parameters [26,32] for both materials are shown in Table 4.

### 3.1. Thermal Model

**Figure 2:**
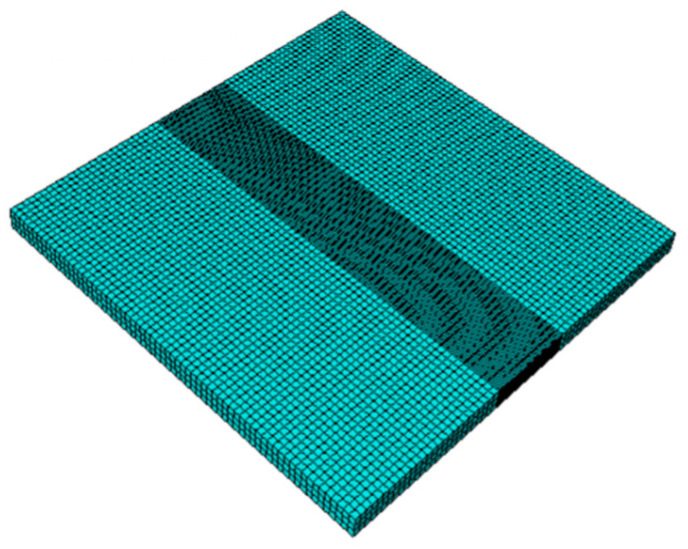
FEM mesh of plates.

The dissimilar aluminum sheets were meshed using DC3D8R three-dimensional elements. There were 18,894 elements and 25,840 nodes in the mesh. Biased meshing was utilized to incorporate a finer mesh surrounding the welded interface and a relatively courser mesh elsewhere, as shown in Figure 2.

A 3D heat transfer element was used for the thermal analysis, and an auto-adapting heat source was employed via a FORTRAN-coded user subroutine. The heat flux is given separately for each case in Table 5.

**Figure 3:**
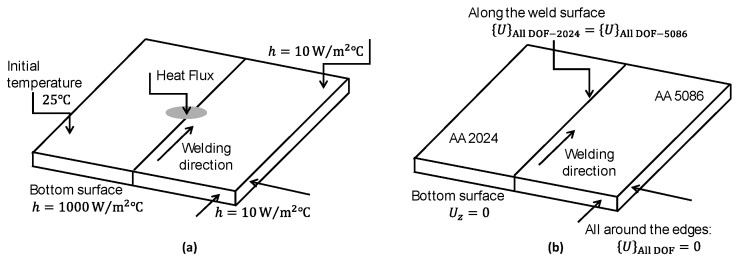
Schematic representation of (**a**) thermal boundary conditions and (**b**) mechanical boundary conditions.

In the thermal model, a convection coefficient of $10\;W/m^{2}\;{}^{\circ}C$ and an ambient temperature of 25 °C at the top and side surfaces are specified, while a larger coefficient of heat transfer of $1000\;W/m^{2}\;{}^{\circ}C$ at the bottom surface is applied. A schematic depiction of the boundary conditions utilized for the thermal analysis is presented in Figure 3a.

**Figure 4:**
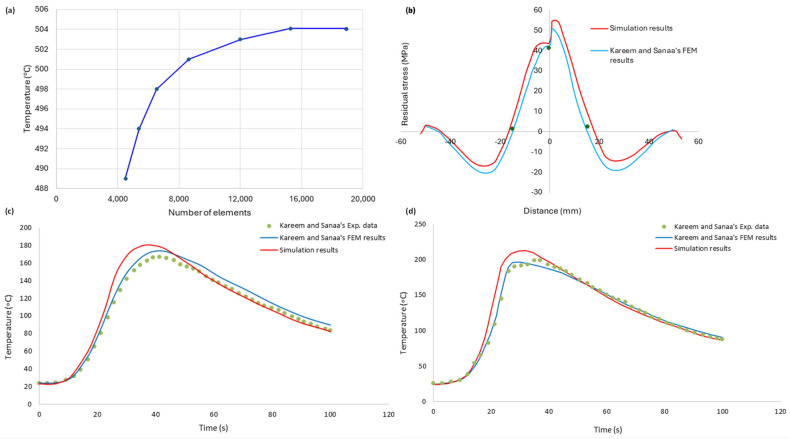
(**a**) Mesh convergence, (**b**) longitudinal residual stress, (**c**) thermal history on the advancing side, and (**d**) thermal history on the retreating side.

A mesh convergence study was carried out to ascertain the reliability of the temperature predictions during welding. The results of the mesh convergence study are shown in Figure 4a.

### 3.2. Mechanical Model

The creation of the mechanical model is the subsequent stage in the thermomechanical analysis. The mechanical model is fed with the temperature distributions obtained in the preceding thermal study. The residual stress caused by welding is estimated using this model. Here, the DC3D8R elements from the heat transfer model analysis are replaced by C3D8R elements. The benefit of utilizing this element type is that its shape, node positions, and coordinates are identical to those of the DC3D8R element—both being reduced integration elements. For the mechanical stress evaluation, the boundary conditions given in Section 2.3 are employed, as shown in the schematic in Figure 3b.

In order to validate the thermomechanical analysis, the obtained results were compared with the findings of Kareem and Sanaa [27]. Their model involved the FSW of two dissimilar aluminum plates, namely AA 2024−T3 and AA 6061−T6 alloys. Figure 4c,d depict the graphical representation of this validation process, demonstrating the comparison of the published results with the simulated thermal histories of this study. The comparisons were made for points 15 mm away from the weld line (Figure 1), where, in the experimental study, thermocouples were placed to measure the temperatures.

Similarly, the mechanical model was validated by comparing the results of the longitudinal residual stress with the published results, as shown in Figure 4b. For the coordinate system used, the longitudinal direction coincides with the x-axis, as shown in Figure 1. The general trend of the model for residual stress prediction is comparable to that of Kareem and Sanaa [27], demonstrating the model’s validity.

## 4. Results and Discussion

This section contains the results for the temperatures and residual stresses generated in the FSW of AA2024−T3 and AA5086−O and focuses on a discussion of these results. Initially, the temperature distribution is presented at the end of the welding pass. Thereafter, the effects of the tool’s traverse and rotational speeds are studied using the temperature profile, which is taken at 15 mm away from the weld centerline on both AA 2024−T3 (advancing side) and AA 5086−O (retreating side), as shown in Figure 1. Finally, the residual stress distribution is presented, along with a discussion of the trends obtained by varying the process parameters.

### 4.1. Temperature Distribution

**Figure 5:**
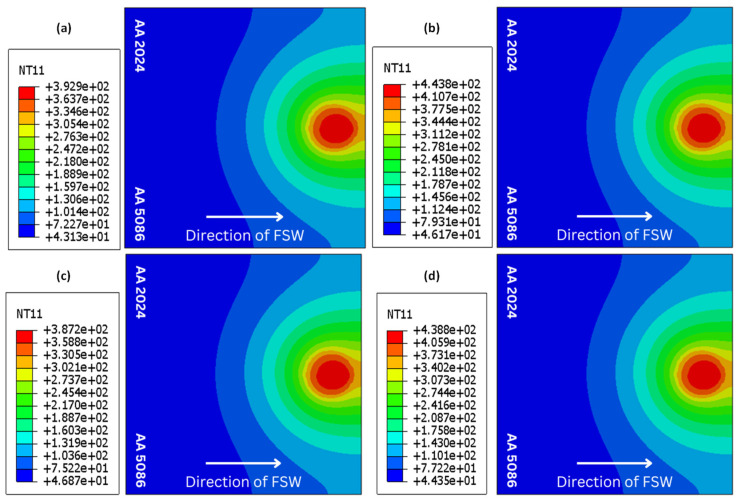
Temperature contours at the end of the welding pass: (**a**) case A, (**b**) case B, (**c**) case C, (**d**) case D.

**Figure 6:**
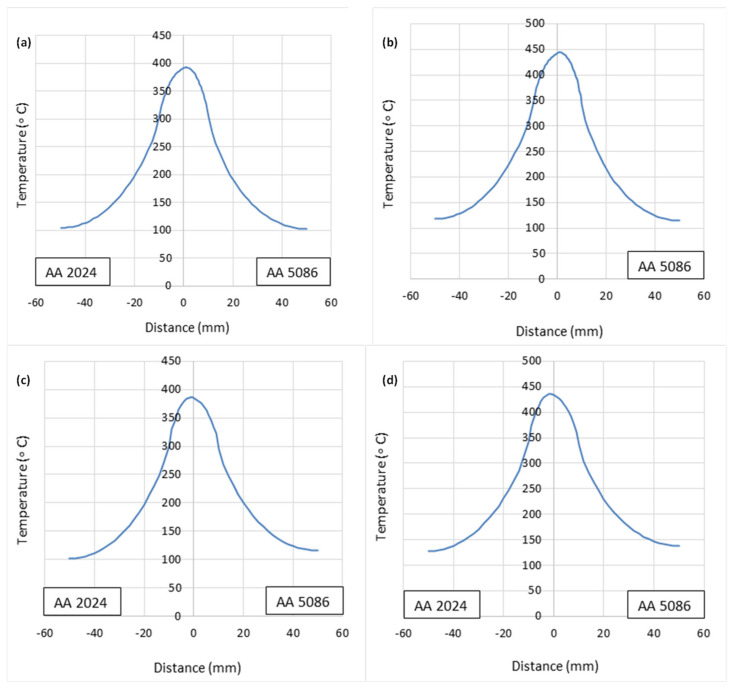
Temperature profile across the width of the workpiece at the end of the welding pass: (**a**) case A, (**b**) case B, (**c**) case C, (**d**) case D.

The temperature contours for all welding configurations at the end of the welding pass are shown in Figure 5. Figure 6 shows the associated temperature profiles at the cross-section through the tool center line. In Figure 6, ‘distance’ refers to the distance from the weld line (perpendicular to the junction of the two plates to be welded). For each configuration, the examination of the thermal history reveals that the temperature distribution around the tool at any given instant is similar to that at the end of the welding pass. The temperatures at the weld line thus achieved are appropriate for the fusion of the dissimilar plates [33].

#### 4.1.1. Effect of Tool Rotational Speed

**Figure 7:**
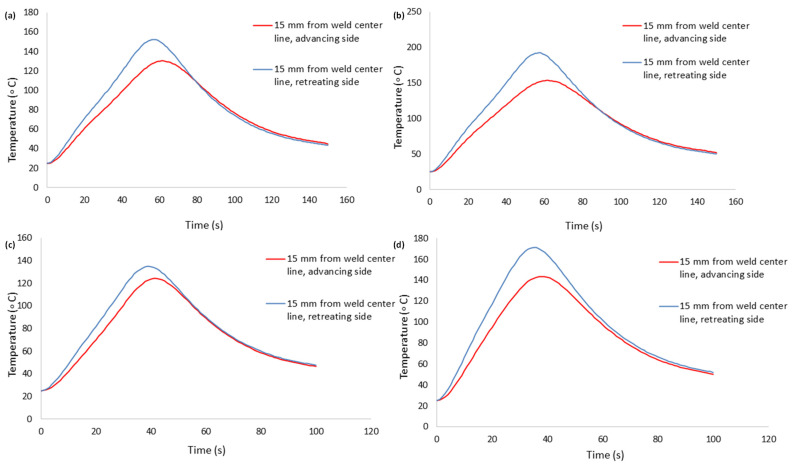
Thermal history at 15 mm from weld center line: (**a**) case A, (**b**) case B, (**c**) case C, (**d**) case D.

The effect of the tool rotational speed on the temperature profile is shown in Figure 7. The amount of heat produced increases with the increase in the tool rotational speed. The higher the tool rotational speed, the greater the heat input, resulting in higher temperatures at various points of the weld sample on both the advancing and retreating sides. Additionally, it is noted from the temperature profiles that the peak temperature in the welded region is more affected by the tool rotational speed. In this numerical study, for the situation of a traverse speed of 40 mm/min, the 400 rpm rotational speed produced a larger peak temperature than that of 300 rpm, as shown in Figure 7a,b. The case of 60 mm/min, as indicated in Figure 7c,d, is the same. Furthermore, it is noticed from the four cases in Figure 7 that the temperature distribution on the retreating side (AA5086−O) is greater as compared to the value of the temperature on the advancing side (AA2024−T3).

#### 4.1.2. Effect of Tool Traverse Speed

The temperature profile is also affected by the tool traverse speed, as seen in Figure 7. It can be observed that a higher traverse speed results in the lowering of the maximum temperature on both the advancing and retreating sides. For the case of a rotational speed of 300 rpm, the 40 mm/min traverse speed produced a larger peak temperature than that of 60 mm/min, as shown in Figure 7a,c. The same trend can be observed in Figure 7b,d. Owing to the faster traverse speed, there is a shorter duration of contact between the spinning tool and the specimen. The duration of contact directly affects how much heat is produced by friction and plastic deformation. It can be seen in all four cases shown in Figure 7 that the temperature distribution on the retreating side is greater than the value on the advancing side.

### 4.2. Residual Stress Distribution

**Figure 8:**
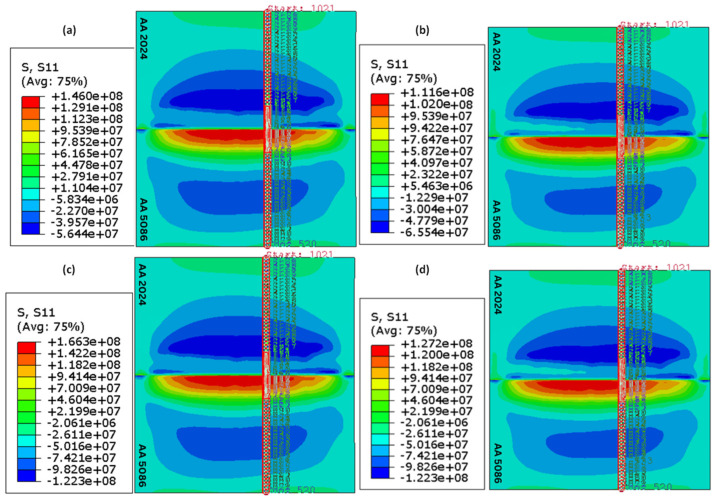
Longitudinal residual stress contours: (**a**) case A, (**b**) case B, (**c**) case C, (**d**) case D.

**Figure 9:**
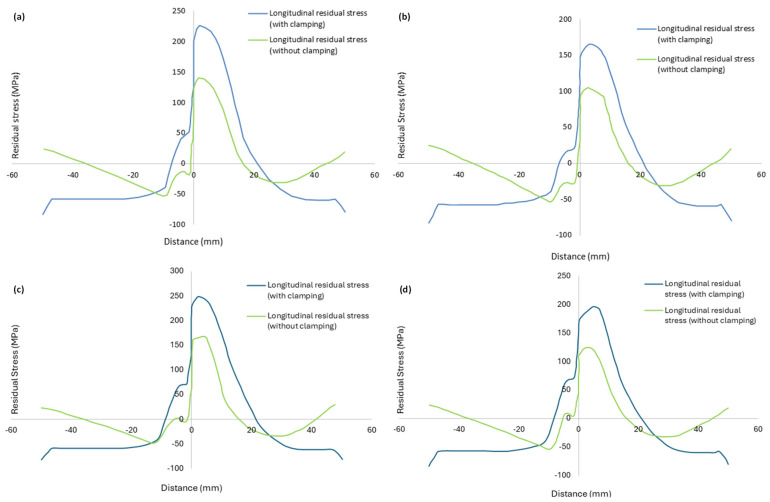
Profile of longitudinal residual stress at x=70 mm: (**a**) case A, (**b**) case B, (**c**) case C, (**d**) case D.

The residual stress distributions obtained as a result of the simulations reveal that the longitudinal residual stress is dominant as compared to the lateral one. The longitudinal residual stress for all welding configurations after the joint had cooled down to room temperature is shown in Figure 8. The contours are depicted for all configurations without clamping. The path along which the profiles of residual stress are recorded is indicated in red in Figure 8. These profiles are presented in Figure 9. In Figure 9, ‘distance’ means the distance from the weld line (perpendicular to the junction of the two plates to be welded) along a specified path (shown in Figure 8).

#### 4.2.1. Effect of Tool Rotational Speed

The spatial distribution of the longitudinal residual stress with and without clamping is shown in Figure 9. All stress profiles correspond to the cross-section shown in red in Figure 8. For a traverse speed of 40 mm/min, a reduction in longitudinal residual stress occurs when the tool rotational speed is raised from 300 rpm to 400 rpm. Consider first the unclamped configurations. For case *A*, the maximum longitudinal residual stress was 140.40 MPa, whereas, for case *B*, the maximum longitudinal residual stress decreased to 105.12 MPa. A similar trend was noticed for a traverse speed of 60 mm/min when the rotational speed was raised from 300 to 400 rpm in cases *C* and *D*, as the maximum longitudinal residual stress decreased from 166.96 MPa for case *C* to 124.64 MPa for case *D*. This is because, for a given traverse speed, a higher rotational speed results in a relatively uniform temperature distribution in the plates, thereby reducing the residual stress.

#### 4.2.2. Effect of Tool Traverse Speed

It is apparent from Figure 9 that raising the traverse speed from 40 mm/min to 60 mm/min causes an increase in the longitudinal residual stress for either rotational speed. Without clamping, for a rotational speed of 300 rpm, the maximum longitudinal stress increases by 18.9%, whereas the increase in the case of 400 rpm is 18.6%. This can be explained by the fact that rapid tool movement produces temperature gradients inside the weld zone, causing different parts to cool at various rates. The findings also indicate that the retreating side has larger residual stress than the advancing side in all four cases. For the clamped cases, the residual stress is higher compared to the unclamped ones.

## 5. Conclusions

A sequentially coupled thermomechanical 3D FEM model was developed to study the temperature distribution and the resulting residual stress generated in the FSW of AA2024−T3 and AA5086−O as a function of the tool traverse and rotational speeds. AA2024−T3 was arranged in this study to be on the advancing side, while AA5086−O was on the retreating side. The model was verified using established experimental and numerical results. This benchmarking validated the use of the sequentially coupled thermomechanical model in determining the temperature distribution and residual stress.

The results show that both the tool rotation and traverse speeds affect the temperature distribution and therefore the peak temperatures reached during the welding process. However, the influence of the tool rotational speed is greater than that of the traverse speed. It has also been demonstrated that the temperatures remain higher on the retreating side (AA5086−O) than the advancing side (AA2024−T3).

The rotational and the traverse speeds also affect the longitudinal residual stress distribution. The magnitude of the maximum residual stress drops as the tool rotational speed increases. The tool traverse speed has the opposite effect. That is, as the traverse speed increases, the maximum residual stress also increases. As with the case of the temperature, the tool rotational speed is more substantial as compared to the tool traverse speed. Furthermore, the maximum residual stress values for both the clamped and unclamped cases are greater on the retreating side (AA5086−O) than the advancing side (AA2024−T3). These trends are attributable to differences in the heat generated, which result in various temperature distributions in a given instance.

## Figures and Tables

**Table 1:** FSW parameters used in the study.

Designation	Rotational Speed (rpm)	Traverse Speed (mm/min)
A	300	40
B	400	40
C	300	60
D	400	60

**Table 2:** Thermal and structural properties of AA 2024−T3.

Material	Density	Temperature	Young’s Modulus	Thermal Expansion	Thermal Conductivity	Specific Heat
	$(kg/m^{3})$	(°C)	(GPa)	$\left(10^{-6}/{}^{\circ}C\right)$	$\left(W/m^{\circ }C\right)$	$\left(J/kg^{\circ }C\right)$
AA 2024−T3	2770	25	72	22	121	875
		37.8	71.9	23	124	895
		93.3	66.5	23.5	128	906
		148.9	63.5	24.5	139	925
		204.4	63	24.8	153	950
		260	57.1	25.3	172	990
		315.6	51.4	25.5	178	1040
		371.1	47.3	26	182	1080
		426.7	44	26.6	190	1110

**Table 3:** Thermal and structural properties of AA 5086−O.

Material	Density	Young’s Modulus		Thermal Expansion		Thermal Conductivity		Specific Heat	
	$\left(\mathrm{kg}/m^{3}\right)$	GPa	T (°C)	$10^{-6}/{}^{\circ}C$	T (°C)	W/m°C	T (°C)	J/kg°C	T (°C)
AA 5086−O	2567	70.0	25	23.8	25	127	25	900	25
		67.8	100	25.5	200	151	250	960	250
		60.7	200	26.8	300	154	300	980	300
		51.0	300	28.9	400	158	400	1020	400
		37.4	400	31.5	500	169	500	1113	500

**Table 4:** Johnson–Cook model constants for AA 2024−T3 and AA 5086−O.

Material	A (MPa)	B (MPa)	N	C	m	$T_{\mathrm{melt}\;}\left({}^{\circ}C\right)$	$T_{\mathrm{room}\;}\left({}^{\circ}C\right)$
AA2024−T3	369	684	0.73	0.0083	1.7	521	25
AA5086−O	170	425	0.42	0.0335	1.225	640	25

**Table 5:** Input heat flux.

Designation	Q (W)	Q/A $\left(W/m^{2}\right)$
A	464.63	$1.479\times 10^{6}$
B	619.54	$1.972\times 10^{6}$
C	464.63	$1.479\times 10^{6}$
D	619.54	$1.972\times 10^{6}$

## Data Availability

The original contributions presented in this study are included in the article. Further inquiries can be directed to the corresponding author.
